# *N*-(2-Chloro-4-nitro­phen­yl)maleamic acid monohydrate

**DOI:** 10.1107/S1600536811052573

**Published:** 2011-12-14

**Authors:** K. Shakuntala, Marek Fronc, B. Thimme Gowda, Jozef Kožíšek

**Affiliations:** a Department of Chemistry, Mangalore University, Mangalagangotri 574 199, Mangalore, India; b Institute of Physical Chemistry and Chemical Physics, Slovak University of Technology, Radlinského 9, SK-812 37 Bratislava, Slovak Republic

## Abstract

The title compound, C_10_H_7_ClN_2_O_5_·H_2_O, crystallizes with a half-mol­ecule each of *N*-(2-chloro-4-nitro­phen­yl)maleamic acid (located on a mirror plane) and water (located on a twofold rotation axis) in the asymmetric unit. The main mol­ecule is planar by symmetry and its conformation is stabilized by an intra­molecular O—H⋯O hydrogen bond. In the crystal, N—H⋯O and O—H⋯O hydrogen bonds link the mol­ecules into a three-dimensional network.

## Related literature

For studies on the effects of substituents on the structures and other aspects of *N*-(ar­yl)-amides, see: Gowda *et al.* (2000 ▶); Prasad *et al.* (2002 ▶); Shakuntala *et al.* (2011 ▶), on *N*-(ar­yl)-methane­sulfonamides, see: Jayalakshmi & Gowda (2004 ▶) on *N*-(ar­yl)-aryl­sulfonamides, see: Shetty & Gowda (2005 ▶) and on *N*-chloro­aryl­sulfonamides, see: Gowda & Kumar (2003 ▶). For modes of inter­linking carb­oxy­lic acids by hydrogen bonds, see: Leiserowitz (1976 ▶).
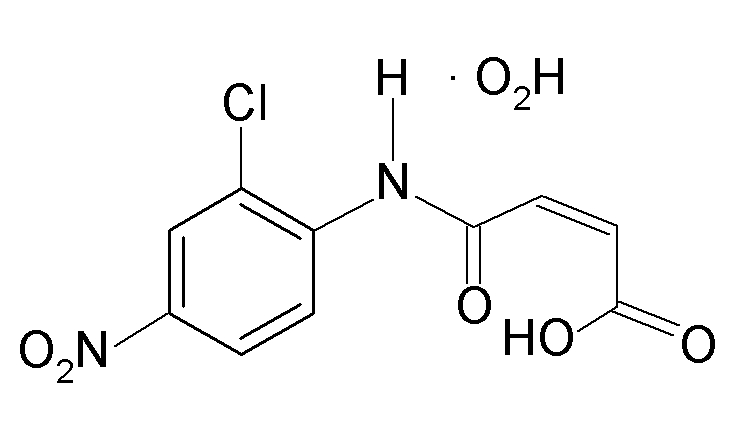


## Experimental

### 

#### Crystal data

C_10_H_7_ClN_2_O_5_·H_2_O
*M* *_r_* = 288.64
Orthorhombic, 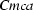

*a* = 6.7499 (2) Å
*b* = 20.3357 (5) Å
*c* = 17.1671 (4) Å
*V* = 2356.42 (11) Å^3^
*Z* = 8
Mo *K*α radiation
μ = 0.35 mm^−1^
*T* = 293 K
0.81 × 0.25 × 0.12 mm

#### Data collection

Oxford Diffraction Xcalibur diffractometer with a Ruby (Gemini Cu) detector
Absorption correction: analytical [*CrysAlis PRO* (Oxford Diffraction, 2009 ▶), based on expressions derived by Clark & Reid (1995 ▶)] *T*_min_ = 0.860, *T*_max_ = 0.965
14309 measured reflections
1310 independent reflections
1131 reflections with *I* > 2σ(*I*)
*R*_int_ = 0.027

#### Refinement

*R*[*F* ^2^ > 2σ(*F* ^2^)] = 0.044
*wR*(*F* ^2^) = 0.128
*S* = 1.07
1310 reflections
118 parameters
1 restraint
H atoms treated by a mixture of independent and constrained refinement
Δρ_max_ = 0.25 e Å^−3^
Δρ_min_ = −0.44 e Å^−3^

### 

Data collection: *CrysAlis PRO* (Oxford Diffraction, 2009 ▶); cell refinement: *CrysAlis PRO*; data reduction: *CrysAlis PRO*; program(s) used to solve structure: *SHELXS97* (Sheldrick, 2008 ▶); program(s) used to refine structure: *SHELXL97* (Sheldrick, 2008 ▶); molecular graphics: *Mercury* (Macrae *et al.*, 2008 ▶) and *DIAMOND* (Brandenburg, 2002 ▶); software used to prepare material for publication: *SHELXL97*, *PLATON* (Spek, 2009 ▶) and *WinGX* (Farrugia, 1999 ▶).

## Supplementary Material

Crystal structure: contains datablock(s) I, global. DOI: 10.1107/S1600536811052573/bt5742sup1.cif

Structure factors: contains datablock(s) I. DOI: 10.1107/S1600536811052573/bt5742Isup2.hkl

Supplementary material file. DOI: 10.1107/S1600536811052573/bt5742Isup3.cml

Additional supplementary materials:  crystallographic information; 3D view; checkCIF report

## Figures and Tables

**Table 1:** Hydrogen-bond geometry (Å, °)

*D*—H⋯*A*	*D*—H	H⋯*A*	*D*⋯*A*	*D*—H⋯*A*
N1—H1*A*⋯O11	0.86	2.50	3.178 (3)	136
O2—H7*W*⋯O1	0.75	1.77	2.515 (3)	171
O11—H11⋯O3^i^	1.05 (1)	2.04 (2)	2.978 (2)	146 (3)

Symmetry code: (i) 
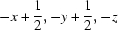
.

**Fig. 1.**
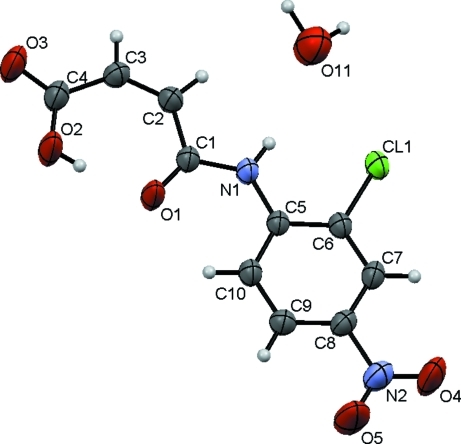
Molecular structure of the title compound showing the atom labelling scheme. Displacement ellipsoids are drawn at the 50% probability level. H atoms are represented as small spheres of arbitrary radii.

**Fig. 2.**
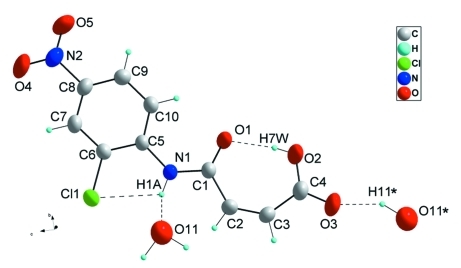
Packing view of the title compound. Molecular chains along a-axis are generated by N–H···O hydrogen bonds which are shown as dashed lines. H atoms not involved in H-bonding have been omitted.

## supplementary crystallographic information

### Comment

The amide moiety is a constituent of many biologically significant compounds. As part of our studies on the substituent effects on the structures and other aspects of *N*-(aryl)-amides (Gowda *et al.*, 2000; Prasad *et al.*, 2002; Shakuntala *et al.*, 2011), *N*-(aryl)-methanesulfonamides (Jayalakshmi & Gowda, 2004), *N*-(aryl)-arylsulfonamides (Shetty & Gowda, 2005) and *N*-chloroarylsulfoamides (Gowda & Kumar, 2003), in the present work, the crystal structure of *N*-(2-chloro-4-nitrophenyl)-maleamic acid monohydrate(I) has been determined (Fig.1).

The conformations of the N—H and the C=O bonds in the amide segment are *anti* to each other. But the conformation of the N—H bond is *syn* to the *ortho*-Cl atom in the phenyl ring, similar to that observed between the N—H bond and *ortho*-methyl group in *N*-(4-Chloro-2-methylphenyl)-maleamic acid (II) (Shakuntala *et al.*, 2011).

In the maleamic acid moiety, the amide C=O bond is *anti* to the adjacent C—H bond, while the carboxyl C=O bond is *syn* to the adjacent C—H bond. The observed rare *anti* conformation of the C=O and O—H bonds of the acid group is similar to that observed in (II). This may be due to the hydrogen bond donated to the amide carbonyl group by the carboxyl group. The C2–C3 bond length of 1.327 (4)Å indicates the double bond character.

The various modes of interlinking carboxylic acids by hydrogen bonds is described elsewhere (Leiserowitz, 1976).

In (I), both the intramolecular O–H···O and N—H···Cl, and intermolecular N–H···O and O–H···O hydrogen bonds have been observed. The packing of molecules linked by intermolecular N–H···O and O–H···O hydrogen bonds into infinite chains is shown in Fig. 2.

### Experimental

The solution of maleic anhydride (0.025 mol) in toluene (25 ml) was treated dropwise with the solution of 2-chloro-4-nitroaniline (0.025 mol) also in toluene (20 ml) with constant stirring. The resulting mixture was stirred for about 30 min and set aside for an additional 30 min at room temperature for the completion of reaction. The mixture was then treated with dilute hydrochloric acid to remove the unreacted 2-chloro-4-nitroaniline. The resultant solid *N*-(2-chloro-4-nitrophenyl)-maleamic acid monohydrate was filtered under suction and washed thoroughly with water to remove the unreacted maleic anhydride and maleic acid. It was recrystallized to constant melting point from ethanol. The purity of the compound was checked and characterized by its infrared spectra.

Prism like colorless single crystals of the title compound used in X-ray diffraction studies were grown in an ethanol solution by slow evaporation (0.5 g in about 30 ml of ethanol) at room temperature.

### Refinement

All hydrogen atoms were placed in calculated positions with C–H distances of 0.93Å and constrained to ride on their parent atoms. Amide and and O—H atoms were seen in difference map and were refined with the N—H distance restrained to 0.86 (1) Å. The *U*_iso_(H) values were set at 1.2 *U*_eq_ (C, N, O).

### Crystal data

**Table:**

C_10_H_7_ClN_2_O_5_·H_2_O	*F*(000) = 1184
*M**_r_* = 288.64	*D*_x_ = 1.627 Mg m^−^^3^
Orthorhombic, *C**m**c**a*	Mo *K*α radiation, λ = 0.71073 Å
Hall symbol: -C 2bc 2	Cell parameters from 4659 reflections
*a* = 6.7499 (2) Å	θ = 2.0–29.4°
*b* = 20.3357 (5) Å	µ = 0.35 mm^−^^1^
*c* = 17.1671 (4) Å	*T* = 293 K
*V* = 2356.42 (11) Å^3^	Prism, colorless
*Z* = 8	0.81 × 0.25 × 0.12 mm

### Data collection

**Table:**

Oxford Diffraction Xcalibur diffractometer with a Ruby (Gemini Cu) detector	1310 independent reflections
Radiation source: fine-focus sealed tube	1131 reflections with *I* > 2σ(*I*)
graphite	*R*_int_ = 0.027
Detector resolution: 10.4340 pixels mm^-1^	θ_max_ = 26.4°, θ_min_ = 4.1°
ω scans	*h* = −8→8
Absorption correction: analytical [*CrysAlis PRO* (Oxford Diffraction, 2009), based on expressions derived by Clark & Reid (1995)]	*k* = −24→25
*T*_min_ = 0.860, *T*_max_ = 0.965	*l* = −21→21
14309 measured reflections	

### Refinement

**Table:**

Refinement on *F*^2^	Secondary atom site location: difference Fourier map
Least-squares matrix: full	Hydrogen site location: inferred from neighbouring sites
*R*[*F*^2^ > 2σ(*F*^2^)] = 0.044	H atoms treated by a mixture of independent and constrained refinement
*wR*(*F*^2^) = 0.128	*w* = 1/[σ^2^(*F*_o_^2^) + (0.0582*P*)^2^ + 3.7851*P*] where *P* = (*F*_o_^2^ + 2*F*_c_^2^)/3
*S* = 1.07	(Δ/σ)_max_ < 0.001
1310 reflections	Δρ_max_ = 0.25 e Å^−^^3^
118 parameters	Δρ_min_ = −0.44 e Å^−^^3^
1 restraint	Extinction correction: *SHELXL97* (Sheldrick, 2008), Fc^*^=kFc[1+0.001xFc^2^λ^3^/sin(2θ)]^-1/4^
Primary atom site location: structure-invariant direct methods	Extinction coefficient: 0.0049 (7)

### Special details

**Table:**

Experimental. CrysAlisPro (Oxford Diffraction, 2009) Analytical numeric absorption correction using a multifaceted crystal model based on expressions derived (Clark & Reid, 1995).
Geometry. All e.s.d.'s (except the e.s.d. in the dihedral angle between two l.s. planes) are estimated using the full covariance matrix. The cell e.s.d.'s are taken into account individually in the estimation of e.s.d.'s in distances, angles and torsion angles; correlations between e.s.d.'s in cell parameters are only used when they are defined by crystal symmetry. An approximate (isotropic) treatment of cell e.s.d.'s is used for estimating e.s.d.'s involving l.s. planes.
Refinement. Refinement of *F*^2^ against ALL reflections. The weighted *R*-factor *wR* and goodness of fit *S* are based on *F*^2^, conventional *R*-factors *R* are based on *F*, with *F* set to zero for negative *F*^2^. The threshold expression of *F*^2^ > σ(*F*^2^) is used only for calculating *R*-factors(gt) *etc*. and is not relevant to the choice of reflections for refinement. *R*-factors based on *F*^2^ are statistically about twice as large as those based on *F*, and *R*- factors based on ALL data will be even larger.

### Fractional atomic coordinates and isotropic or equivalent isotropic displacement parameters (Å^2^)

**Table:**

	*x*	*y*	*z*	*U*_iso_*/*U*_eq_	
C1	0.0000	0.38088 (14)	0.11375 (16)	0.0392 (7)	
C2	0.0000	0.31306 (15)	0.08336 (18)	0.0456 (8)	
H2A	0.0000	0.2800	0.1208	0.055*	
C3	0.0000	0.29270 (15)	0.00997 (17)	0.0450 (8)	
H3A	0.0000	0.2472	0.0048	0.054*	
C4	0.0000	0.32802 (18)	−0.06507 (19)	0.0546 (9)	
C5	0.0000	0.44114 (13)	0.23898 (16)	0.0350 (6)	
C6	0.0000	0.43336 (14)	0.32018 (16)	0.0365 (6)	
C7	0.0000	0.48666 (15)	0.36958 (17)	0.0435 (7)	
H7A	0.0000	0.4810	0.4233	0.052*	
C8	0.0000	0.54865 (15)	0.33704 (18)	0.0440 (8)	
C9	0.0000	0.55845 (14)	0.25794 (19)	0.0425 (7)	
H9A	0.0000	0.6008	0.2377	0.051*	
C10	0.0000	0.50500 (15)	0.20906 (17)	0.0405 (7)	
H10A	0.0000	0.5114	0.1554	0.049*	
Cl1	0.0000	0.35546 (4)	0.36046 (4)	0.0487 (3)	
N1	0.0000	0.38484 (12)	0.19242 (14)	0.0431 (6)	
H1A	0.0000	0.3480	0.2169	0.052*	
N2	0.0000	0.60563 (15)	0.38903 (18)	0.0647 (9)	
O1	0.0000	0.42972 (11)	0.07268 (12)	0.0671 (9)	
O2	0.0000	0.39196 (13)	−0.06678 (14)	0.0886 (12)	
H7W	0.0000	0.4070	−0.0267	0.133*	
O3	0.0000	0.29773 (15)	−0.12558 (15)	0.0864 (11)	
O4	0.0000	0.59696 (16)	0.45824 (16)	0.1041 (14)	
O5	0.0000	0.65986 (14)	0.35994 (18)	0.1049 (15)	
O11	0.2500	0.26168 (15)	0.2500	0.0931 (11)	
H11	0.319 (5)	0.2243 (11)	0.2174 (17)	0.112*	

### Atomic displacement parameters (Å^2^)

**Table:**

	*U*^11^	*U*^22^	*U*^33^	*U*^12^	*U*^13^	*U*^23^
C1	0.0580 (18)	0.0330 (15)	0.0266 (13)	0.000	0.000	−0.0002 (11)
C2	0.075 (2)	0.0294 (15)	0.0328 (15)	0.000	0.000	0.0028 (12)
C3	0.070 (2)	0.0306 (15)	0.0349 (16)	0.000	0.000	−0.0042 (12)
C4	0.089 (3)	0.0443 (19)	0.0306 (16)	0.000	0.000	−0.0048 (14)
C5	0.0481 (16)	0.0313 (13)	0.0257 (13)	0.000	0.000	0.0002 (10)
C6	0.0487 (16)	0.0328 (14)	0.0279 (14)	0.000	0.000	0.0032 (11)
C7	0.063 (2)	0.0418 (17)	0.0259 (13)	0.000	0.000	−0.0025 (12)
C8	0.062 (2)	0.0360 (16)	0.0341 (15)	0.000	0.000	−0.0082 (12)
C9	0.0593 (19)	0.0311 (14)	0.0371 (16)	0.000	0.000	0.0002 (12)
C10	0.0587 (18)	0.0357 (15)	0.0270 (14)	0.000	0.000	0.0020 (11)
Cl1	0.0793 (6)	0.0367 (4)	0.0301 (4)	0.000	0.000	0.0070 (3)
N1	0.0757 (18)	0.0288 (12)	0.0248 (11)	0.000	0.000	0.0015 (9)
N2	0.108 (3)	0.0425 (17)	0.0436 (17)	0.000	0.000	−0.0118 (13)
O1	0.144 (3)	0.0313 (12)	0.0258 (11)	0.000	0.000	0.0009 (9)
O2	0.197 (4)	0.0424 (15)	0.0262 (12)	0.000	0.000	0.0001 (10)
O3	0.166 (3)	0.0607 (17)	0.0327 (13)	0.000	0.000	−0.0123 (12)
O4	0.213 (4)	0.0621 (19)	0.0368 (15)	0.000	0.000	−0.0167 (13)
O5	0.218 (5)	0.0353 (14)	0.0618 (19)	0.000	0.000	−0.0111 (13)
O11	0.145 (3)	0.0680 (19)	0.0664 (19)	0.000	0.021 (2)	0.000

### Geometric parameters (Å, °)

**Table:**

C1—O1	1.218 (4)	C6—Cl1	1.728 (3)
C1—N1	1.353 (4)	C7—C8	1.379 (4)
C1—C2	1.474 (4)	C7—H7A	0.9300
C2—C3	1.326 (4)	C8—C9	1.372 (4)
C2—H2A	0.9300	C8—N2	1.463 (4)
C3—C4	1.475 (4)	C9—C10	1.373 (4)
C3—H3A	0.9300	C9—H9A	0.9300
C4—O3	1.208 (4)	C10—H10A	0.9300
C4—O2	1.301 (4)	N1—H1A	0.8600
C5—C10	1.397 (4)	N2—O4	1.201 (4)
C5—N1	1.396 (4)	N2—O5	1.211 (4)
C5—C6	1.403 (4)	O2—H7W	0.7531
C6—C7	1.376 (4)	O11—H11	1.052 (3)
			
O1—C1—N1	122.0 (3)	C6—C7—H7A	121.0
O1—C1—C2	123.9 (3)	C8—C7—H7A	121.0
N1—C1—C2	114.1 (3)	C9—C8—C7	122.2 (3)
C3—C2—C1	128.9 (3)	C9—C8—N2	119.3 (3)
C3—C2—H2A	115.5	C7—C8—N2	118.5 (3)
C1—C2—H2A	115.5	C10—C9—C8	119.3 (3)
C2—C3—C4	132.7 (3)	C10—C9—H9A	120.3
C2—C3—H3A	113.7	C8—C9—H9A	120.3
C4—C3—H3A	113.7	C9—C10—C5	120.8 (3)
O3—C4—O2	119.4 (3)	C9—C10—H10A	119.6
O3—C4—C3	120.2 (3)	C5—C10—H10A	119.6
O2—C4—C3	120.4 (3)	C1—N1—C5	128.3 (3)
C10—C5—N1	123.5 (3)	C1—N1—H1A	115.9
C10—C5—C6	118.1 (3)	C5—N1—H1A	115.8
N1—C5—C6	118.4 (2)	O4—N2—O5	122.8 (3)
C7—C6—C5	121.6 (3)	O4—N2—C8	119.2 (3)
C7—C6—Cl1	118.4 (2)	O5—N2—C8	118.0 (3)
C5—C6—Cl1	120.1 (2)	C4—O2—H7W	112.6
C6—C7—C8	118.1 (3)		
			
O1—C1—C2—C3	0.0	C7—C8—C9—C10	0.000 (1)
N1—C1—C2—C3	180.0	N2—C8—C9—C10	180.0
C1—C2—C3—C4	0.0	C8—C9—C10—C5	0.0
C2—C3—C4—O3	180.0	N1—C5—C10—C9	180.0
C2—C3—C4—O2	0.0	C6—C5—C10—C9	0.0
C10—C5—C6—C7	0.0	O1—C1—N1—C5	0.0
N1—C5—C6—C7	180.0	C2—C1—N1—C5	180.0
C10—C5—C6—Cl1	180.0	C10—C5—N1—C1	0.0
N1—C5—C6—Cl1	0.0	C6—C5—N1—C1	180.0
C5—C6—C7—C8	0.0	C9—C8—N2—O4	180.0
Cl1—C6—C7—C8	180.0	C7—C8—N2—O4	0.000 (1)
C6—C7—C8—C9	0.000 (1)	C9—C8—N2—O5	0.000 (1)
C6—C7—C8—N2	180.0	C7—C8—N2—O5	180.0

### Hydrogen-bond geometry (Å, °)

**Table:**

*D*—H···*A*	*D*—H	H···*A*	*D*···*A*	*D*—H···*A*
N1—H1A···O11	0.86	2.50	3.178 (3)	136.
O2—H7W···O1	0.75	1.77	2.515 (3)	171.
O11—H11···O3^i^	1.05 (1)	2.04 (2)	2.978 (2)	146 (3)

Symmetry codes: (i) −*x*+1/2, −*y*+1/2, −*z*.
